# Short Report: Barriers and facilitators to parents' implementation of a transdiagnostic eHealth sleep intervention for children with neurodevelopmental disorders

**DOI:** 10.3389/frsle.2023.1143281

**Published:** 2023-06-01

**Authors:** Anastasija Jemcov, Lindsay Rosenberg, Kim Tan-MacNeill, Isabel M. Smith, Shelly K. Weiss, Penny Corkum

**Affiliations:** ^1^Corkum LABS, Department of Psychology and Neuroscience, Faculty of Science, Dalhousie University, Halifax, NS, Canada; ^2^Autism Research Centre, Department of Pediatrics, Faculty of Medicine, Dalhousie University, Halifax, NS, Canada; ^3^IWK Health Centre, Halifax, NS, Canada; ^4^SickKids, Department of Pediatrics, Faculty of Medicine, University of Toronto, Toronto, ON, Canada; ^5^Department of Psychiatry, Faculty of Medicine, Dalhousie University, Halifax, NS, Canada

**Keywords:** sleep, behavioral insomnia, neurodevelopmental disorders, eHealth intervention, qualitative analysis, implementation

## Abstract

**Background:**

Insomnia is highly prevalent in children diagnosed with neurodevelopmental disorders (NDDs) and has negative effects on physical and mental health and wellbeing. Lack of evidence-based intervention programs and barriers to treatment (e.g., time/cost) reduce treatment access. To address these problems, the possibility was explored of modifying the Better Nights, Better Days intervention for typically developing (TD) children (BNBD-TD) to make it appropriate for children with NDD.

**Aims:**

The current study's aim was to examine qualitative data from exit interviews conducted during a usability study. Parents of children with NDD used BNBD-TD and reported on barriers and facilitators experienced while implementing the intervention.

**Methods/procedures:**

Participants were 15 Canadian parents of children aged 4 to 10 years who were formally diagnosed with an NDD. Parents implemented the BNBD-TD intervention with their children and participated in a semi-structured exit interview to provide perspectives on their user experience.

**Results:**

Based on an inductive thematic analysis, key facilitators included increased self-efficacy, positive outcomes for the family (e.g., improved sleep problems, parent validation), improved sleep related beliefs/attitudes, and increased motivation. Key barriers included time challenges, struggles when trying to improve sleep problems, and psychosocial factors with negative effects on implementation (e.g., burnout, stress, and/or exhaustion).

**Conclusions/implications:**

Barriers and facilitators identified resulted in recommendations to include more program supports, including helping parents to plan for implementation success.

## Introduction

Insomnia is highly prevalent in children who have been diagnosed with a neurodevelopmental disorder (NDD), with up to 86% experiencing insomnia symptoms (i.e., sleep problems such as difficulties falling/staying asleep and night and/or early morning awakenings; Didden and Sigafoos, 2001; American Psychiatric Association., 2013). Unfortunately, parents of children diagnosed with an NDD experience significant difficulty with accessing evidence-based insomnia intervention as few effective intervention programs exist, and barriers such as long wait times and costs to attend appointments (e.g., childcare, parking) reduce access to those that do (Tan-MacNeill et al., 2020a). Therefore, there is a strong need for an accessible evidence-based sleep intervention for children with NDDs to address such barriers.

One such way to deliver this type of intervention in an accessible manner is through an eHealth program. eHealth refers to the use of digital platforms to provide healthcare interventions, such as behavioral therapy. Benefits of eHealth intervention include increased accessibility, and the ability to deliver interventions in a cost-effective manner. Additionally, these interventions allow for increased flexibility in terms of timing and location, which can be particularly beneficial for those who have busy schedules or live in remote areas. Overall, eHealth has shown great promise in improving access to mental health care (Leblanc et al., 2020).

To our knowledge, there are currently no eHealth transdiagnostic evidence-based sleep interventions developed specifically for children with NDDs (i.e., a single intervention that addresses sleep across children with various NDD diagnoses; Tan-MacNeill et al., 2021). Our research team has conducted a series of studies to determine whether the Better Nights, Better Days for Typically Developing (TD) Children (BNBD-TD) eHealth behavioral sleep intervention could be modified for parents of children with NDD. A detailed description of the BNBD-TD program is provided in the “Intervention” section of this manuscript.

In this research, we focused on four NDD diagnoses, Attention Deficit/Hyperactivity Disorder (ADHD), Autism Spectrum Disorder (ASD), Fetal Alcohol Spectrum Disorder (FASD), and Cerebral Palsy (CP). Thus far, we have demonstrated evidence suggesting that a transdiagnostic sleep intervention may address insomnia in children with NDD (Ali et al., 2018; Rigney et al., 2018; Tan-MacNeill et al., 2020a). This evidence has been collected from the perspectives of sleep experts (Ali et al., 2018), as well as from parents of children with NDD (Tan-MacNeill et al., 2020a). In the systematic review by Rigney et al. (2018), we concluded that behavioral sleep interventions used for children with NDD were similar across NDD diagnoses and to interventions for TD children (i.e., psychoeducation, healthy sleep practices, graduated extinction, and reinforcement). In the Delphi study conducted by Ali et al. (2018), sleep experts (i.e., 12 clinicians/researchers with pediatric sleep expertise) all provided similar recommendations across NDDs regarding what would be important/relevant to include in a sleep intervention for children with NDD. In the Tan-MacNeill et al. (2020a) barriers and facilitators study, parents of children with NDDs reported finding similar sleep strategies to be helpful across diagnoses (e.g., use of bedtime routines, healthy sleep habits, etc.; Tan-MacNeill et al., 2020a). Taken together, these three studies provide strong evidence for the feasibility and potential acceptability and effectiveness of a transdiagnostic eHealth insomnia intervention.

The fourth study in this series was a usability study in which parents of children with the above-mentioned NDDs used the original BNBD-TD program and provided feedback about the usability of the program. Results showed the intervention to be highly usable, feasible, and acceptable (Tan-MacNeill et al., 2020b). Upon completion of the usability study, parents were invited to participate in an exit interview to evaluate the factors that contributed to implementation success and challenges (i.e., factors that could affect parents' ability to use/carry out the intervention).

Here we present a short report of the findings from the exit interviews, answering the research question: What implementation factors (i.e., both barriers and facilitators) affect parents' ability to successfully implement BNBD-TD for their children with NDDs?

## Method

### Participants

Of the 20 parent participants in the usability study (Tan-MacNeill et al., 2020b), 15 completed an exit interview. They were all Canadian parents with a 4- to 10-year-old child formally diagnosed by a physician/psychologist with a primary diagnosis of ADHD (*n* = 4), ASD (*n* = 6), CP (*n* = 1), or FASD (*n* = 4). These children met research criteria for insomnia which was determined by the participants' score on the behavioral insomnia questionnaire (Tan-MacNeill et al., 2020b). Their parents participated in the usability study and completed all sessions of BNBD-TD. Details regarding parent and child demographic characteristics (e.g., income, age) are provided in Table 1. Inclusion criteria were that participants must be the primary caregiver of the child aged 4 to 10 years who has a diagnosis of ADHD, ASD, CP, or FASD given by a physician or psychologist, live in Canada, have regular access to high-speed internet, email, and phone/web-camera, be fluent in English, have insomnia, and attend school or preschool. Exclusion criteria were that the participant wished to bed-share/co-sleep with child, have a child diagnosed with a sleep disorder other than insomnia (e.g., sleep apnea) or other significant medical disorder that interferes with sleep, sleep-breathing problems (as determined by the Pediatric Sleep Questionnaire), mental health disorder that required/currently requires hospitalization/residential care (excluding emergency room visits), non-ambulatory/not able to turn self over in bed, functional impairment in adaptive skills as determined by caregiver report (e.g., not dry during the day, not able to self-feed, cannot participate in dressing self), child is currently on anti-epileptic and/or psychotropic medication (excluding stimulant medication), and over the counter/natural health medications (except melatonin to treat child's sleep problems) (Tan-MacNeill et al., 2020b).

**Table 1:** Demographic and descriptive information.

	**Total**
	**(*****N*** = **15)**
**Participant demographics**	
Participants' relationship to child	
Biological mother	10
Adoptive parent	5
Participants' relationship status	
Common-law relationship	3
Legally married	10
Other	2
Participants' current employment status	
Full time	6
Part time	6
Other	3
Estimated household income^*^	
$79,9999 and under	6
$80,000–$124,9999	3
$125,000 and over	5
**Child demographics**	
Child gender	
Male	14
Female	1
Child mean age in years (SD)	9.87 (0.98)
Child's ethnic or cultural heritage	
White/Caucasian	13
Other	2

^*^Indicates missing data for one participant for Estimated Household Income.

### Intervention

BNBD-TD is a self-guided sleep intervention that provides parents with evidence-based strategies, personalized content, and age-specific information for TD children aged 1–10 years experiencing insomnia symptoms; (Corkum et al., 2019). This intervention provides parents with support and guidance delivered through an online platform and has been shown to be effective in improving TD children's sleep, reducing parental stress, and improving family quality of life (Corkum et al., 2019). The intervention consists of five sessions that build upon each other. The first session introduces the intervention, including information about how sleep works and how sleep problems develop in children. In session two, parents learn about healthy sleep habits and practices to help improve their child's sleep. Session three focuses on strategies for parents to help their child settle to sleep more independently at bedtime. In session four, parents receive strategies on how to help their child settle after night wakings, settle for nap time, and manage early morning awakenings. Session five provides parents with an overview of their goals and progress and provides tools on how to plan for the future. Finally, there is a reward center that allows parents to develop a reward program for their child as they complete the program. During the one-week interval between each session, parents track their child's sleep with a sleep diary, which records information such as when they fell asleep, the number of night wakings, when they woke up, and other relevant information. There is also a roadblock section in which parents can search for suggestions on how to address challenges with implementing the strategies.

### Procedure

As part of a larger usability study, parents were given access to the BNBD-TD intervention. After completing the program, parents who opted to participate in the semi-structured exit interview were asked 20-open ended questions focused on their general impressions of the program, implementation, broadening perspective to other families of children with NDD, and concluding questions. Of these 20 questions, the 9 that were focused on implementation were analyzed, and 11 were reported in Tan-MacNeill et al. (2020b). These interviews took place between September of 2017 and February of 2018. Interviews were scheduled approximately one-week after completing the BNBD-TD intervention. The interview guide was developed based on themes identified in parent focus groups conducted by Tan-MacNeill et al. (2020a), such as support, treatment adherence, and sleep-related knowledge and beliefs. These themes represented important theoretical areas of interest to determine the barriers and facilitators that are crucial for implementation (Atkins et al., 2017). Lastly, the current study adhered to the consolidated criteria for reporting qualitative research (COREQ; Tong et al., 2007), please see our supplemental document.

### Data analytic approach

The current study used an inductive thematic analysis approach based on Braun and Clarke (2006) step-by-step guide. This type of approach involves identifying themes that may not be related to the specific questions that were asked of participants, and is driven by the data itself as opposed to pre-existing categories or ideas. In contrast, a theoretical approach involves using pre-existing categories or ideas to guide investigation. For example, from a specific model or framework of implementation (e.g., TDF, the Consolidated Framework for Implementation Research [CFIR]; Damschroder et al., 2009).

The specific steps of the qualitative analysis involved the researchers first becoming familiar with the data by reading all interview transcripts and noted their initial impressions. The second step was generating subthemes, which were then grouped into organizing themes (i.e., barriers and facilitators). Overall inter-rater reliability was 91%. To resolve disagreements in coding (9%) the senior researcher (PC) was consulted.

## Results

### Facilitators to implementation

For facilitators to implementation, five themes were identified and among these were eight subthemes: 1. Intervention use positively affecting family [subthemes: sleep problems improved, parent validation], 2. Increased parental self-efficacy for treating child's sleep problems [subthemes: improved ability to treat sleep problems, parent empowerment after using the intervention], 3. Improved sleep-related beliefs/attitudes [subthemes: sleep beliefs improved following sleep psychoeducation/additional information], 4. Doing something that helps your child as a motivating factor [subthemes: motivated to help their child sleep, ready to make changes to child's sleep routines], and 5. Using the intervention as a motivating factor [subthemes: intervention use/progress motivates further use].

Overall, the majority of parents were motivated to implement the intervention to help their children and also expressed that they felt ready to make changes to their children's sleep routines, with 10 out of 15 reporting this (i.e., 67%). All parents in the study reported feeling validated by the intervention, with comments such as, “*the program helped my family see that of course I feel frustrated, this is a big problem, and a big enough problem that other parents would need a program like this too because sometimes you can feel alone with it”*). Additionally, all parents reported that the intervention was helpful in treating their children's sleep problems, with comments such as, “*it has made a huge difference in our family especially with the bedtime routine, this is the first intervention that actually worked for us*”. The majority of parents (i.e., 13/15) also reported that the intervention enhanced their self-efficacy, with comments such as, “*I now have the tools available to me and I can pretty much do anything! I sound like superwoman”*. Furthermore, 80% of parents (i.e., 12/15) reported that the intervention positively influenced sleep-related beliefs/attitudes, with comments such as, “*honestly [the parent's sleep knowledge] before? I didn't have a clue, perhaps because of misinformation. What I like is this program is devoted to sleep so I would say that I am more knowledgeable about it now”*.

### Barriers to implementation

Three barriers to implementation were identified, with seven subthemes: 1. Time challenges when using the intervention [subthemes: schedule/routine transition periods, difficulty finding time to complete intervention, environmental changes/extenuating circumstances], 2. Struggle when trying to improve sleep problems [subthemes: needing patience to see consistent results, continued sleep challenges as de-motivating when using strategies], 3. Psychosocial factors that negatively impact ability to implement the intervention [subthemes: parent burnout/stress/exhaustion, difficulty for whole family to adapt to intervention strategies].

Overall, results demonstrated that 73% of parents (i.e., 11/15) experienced time challenges, with comments such as, “*I used it [intervention] in the month of December [referring to the holidays], I think some other time of the year where there's not much going on would have been easier*”. Results also demonstrated 73% of parents (i.e., 11/15) had difficulty with implementing the intervention itself (e.g., “*just the increased stress of his resistance to it [intervention]. We spend quite a bit of time fighting, it was a struggle”*). Lastly, 47% of parents (i.e., 7/15) experienced psychosocial difficulty, with comments such as, “*we're all exhausted [the family] and don't have the energy to help”*, as well as, “*you have your own child and you have your family expectations too, so others in the family also have to stop watching electronics in the evening*”).

## Discussion

This goal of this study was to identify what factors parents of children with NDD identified as barriers and facilitators to the successful implementation of the BNBD-TD intervention. Generally, parents were motivated to implement the intervention, and those families that experienced success while implementing the intervention (e.g., child sleep problems improved) experienced positive outcomes for their families such as feelings of validation, improved self-efficacy and sleep-related beliefs. On the contrary, those families experiencing challenges with implementing the intervention (i.e., time challenges, difficulties having their families adapt to intervention strategies) reported concurrently experiencing psychosocial difficulties such as stress/burnout/exhaustion. Taken together, the intervention was more easily implemented by parents who could see positive outcomes for their family and child. This highlights the importance of addressing the barriers identified to successful implementation. These results support further research exploring how to best support families experiencing these barriers (e.g., addition of coach support to the program), and/or whether planning should occur ahead of intervention use (e.g., to identify the appropriate time and/or supports they would need for successful implementation). As such, taking into consideration interpersonal processes (e.g., social support, parent stress/burnout) as well as environmental influences (e.g., timing of implementation) ahead of intervention use may promote successful implementation.

We note limitations of the research. Although a sample size of fifteen is within the recommendations for qualitative research (Ando et al., 2014), we could not make comparisons between NDD diagnostic groups given the small numbers. Also, parents interviewed for this research had been successful in completing the BNBD-TD intervention; as such, these data may not represent all barriers. Additionally, the majority of the current study's sample was comprised of Caucasian participants living in high-income homes. This is consistent with previous sleep intervention research in which the samples were primarily White and from predominantly White countries with moderate to high education level (Schwichtenberg et al., 2019). Future research should aim to involve racially and ethnically minoritized families, as well as those families living in lower income households.

Overall, this research has been translated in the changes made to develop the BNBD-TD version that has been modified for NDD populations (BNBD-NDD). The modifications include diagnostic specific information that was added for ADHD, ASD, FASD, and CP. Also, more time was given for parents within each session to try out strategies, sessions were broken down into smaller sets to support parents with busier schedules, and the sleep diary was made easier to understand, and more smartphone friendly. These modifications help to address the barriers identified in the current study such as difficulty implementing the intervention, time challenges, and psychosocial factors negatively impacting ability to implement the intervention.

In conclusion, if the intervention works for the family, this translates to parents having improved self-efficacy, positive impacts to the family (i.e., child's sleep problems improved, parent validation), improved sleep-related beliefs and attitudes, and increased motivation. However, for parents who did not experience success, stress, burn-out, and/or exhaustion may have contributed. These results identified the importance of supporting parents and planning ahead to ensure the success of the intervention and positive outcomes for the whole family.

## Data availability statement

The datasets presented in this article are not readily available because of ethical restrictions. Requests to access the datasets should be directed to the corresponding author.

## Ethics statement

The studies involving human participants were reviewed and approved by IWK Children's Hospital Research Ethics Board Halifax, NS, Canada. The patients/participants provided their written informed consent to participate in this study.

## Author contributions

AJ contributed to the conceptual development of this research, research ethics process, data collection, development of the research analytic approach, analysis, and preparation of the written manuscript. LR contributed to data analysis and review of the written manuscript. KT-M contributed to the conceptual development of this research, research ethics process, data collection, and review of the written manuscript. IS contributed to the conceptual development of this research, research ethics process, conceptual development of this research, and review of the written manuscript. SW contributed to the research grant preparation which funded this project, and review of the written manuscript. PC contributed to the conceptual development of this research, research grant preparation which funded this project, development of the research analytic approach, supervision of all trainees involved, research ethics process, analysis, and review of the written manuscript. All authors contributed to the article and approved the submitted version.
