# Supplementary material for: Short Report: Barriers and facilitators to parents' implementation of a transdiagnostic eHealth sleep intervention for children with neurodevelopmental disorders
Source: Front Sleep. 2023 Jun 1;2:1143281. doi: 10.3389/frsle.2023.1143281 (PMC12713910; doi:10.3389/frsle.2023.1143281)
Supplement: Supplementary file 1 [file Table_1.docx]

| Supplemental Table 1. Consolidated criteria for reporting qualitative studies (COREQ) checklist with answers | | |
| --- | --- | --- |
|  | Guide questions/description | Answer |
| **Domain 1 Research team and reflexivity** | | |
| Personal Characteristics |  |  |
| 1. Interviewer/facilitator | Which author/s conducted the interviews? | Tan-MacNeill & Jemcov |
| 1. Credentials | What were the researcher’s credentials at the time of the interviews? | PhD Candidates |
| 1. Occupation | What was their occupation at the time of the study? | Student |
| 1. Gender | Was the researcher male or female | Both interviewers, and coders involved with the analyses are female |
| 1. Experience and training | What experience or training did the researcher have? | Interviewers and coders were all trained in a clinical psychology PhD program as researcher-clinicians |
| Relationship with participants |  |  |
| 1. Relationship established | Was a relationship established prior to study commencement? | No |
| 1. Participant knowledge of interviewer | What did the participants know about the researcher? *e.g., personal goals, reasons for doing the research* | Researcher’s background (role as graduate student, primary area of research was autism/neurodevelopmental disorders and sleep, location of graduate studies, university affiliation) and involvement with ‘Better Nights, Better Days’ project modification |
| 1. Interviewer characteristics | What characteristics were reported about the interviewer/facilitator? *e.g., bias assumptions, reasons and interests in the research topic* | No personal biases or characteristics reported; participants may have surmised interviewer’s ethnicity from visual features and last name; reason for research topic was reported as helping to modify the original ‘Better Nights, Better Days’ project into an intervention for children with neurodevelopmental disorders |
| **Domain 2: Study design** | | |
| Theoretical framework |  |  |
| 1. Methodological orientation and theory | What methodological orientation was stated to underpin the study? *e.g.,* *grounded theory, discourse analysis, ethnography, phenomenology, content analysis* | The methodological orientation utilized was content analysis |
| Participant selection |  |  |
| 1. Sampling | How were participants selected? *e.g., purposive, convenience, snowball* | A combination of random and purposive sampling |
| 1. Method of approach | How were participants approached? *e.g., face-to-face, telephone, mail, email* | For study recruitment, participants who had completed the usability study were approached via email to participate in the exit interview |
| 1. Sample size | How many participants were in the study? | 15 (Autism Spectrum Disorder [ASD] = 6, Attention Deficit/Hyperactivity Disorder [ADHD] = 4, Fetal Alcohol Spectrum Disorder [FASD] = 4, Cerebral Palsy [CP] = 1) |
| 1. Non-participation | How many people refused to participate or dropped out? Reasons? | Of the 20 parents participating in the Usability study, 15 participated in the exit interview. Primary reasons for not participating in the interview were not responding to researcher attempts to schedule/book the exit interview or non-response after not completing an earlier module of the intervention, or dropping out of the study at an earlier point |
| Setting |  |  |
| 1. Setting of data collection | Where was the data collected? | Data was collected online using Blackboard Collaborate |
| 1. Presence of non-participants | Was anyone else present beside the participants and researchers? | No one else was present aside from both of the researchers |
| 1. Description of sample | What are the important characteristics of the sample? | The sample consisted of parents/caregivers of young children diagnosed with a neurodevelopmental disorder with sleep problems. For further details about the sample, please see the demographic characteristics listed in Table 1 |
| Data collection |  |  |
| 1. Interview guide | Were questions, prompts, guides, provided by the authors? Was it pilot tested? | The interview guide is available as a supplemental document. The interview guide was pilot tested among the researchers (Tan-MacNeill & Jemcov) and supervisors (Corkum & Smith) |
| 1. Repeat interviews | Were repeat interviews carried out? If yes, how many? | No repeat interviews were carried out |
| 1. Audio/visual recording | Did the research use audio or visual recording to collect the data? | Only audio recordings were used in order to best protect participant privacy |
| 1. Field notes | Were field notes made during and/or after the interview? | The interviews were audio-recorded and transcribed at a later time. No notes were taken during the interview |
| 1. Duration | What was the duration of the interview? | The interviews ranged from 20:10 to 38:52 (minutes:seconds) in length |
| 1. Data saturation | Was data saturation discussed? | Yes, among the researchers (Tan-MacNeill & Jemcov), and supervisors (Corkum & Smith) |
| 1. Transcripts returned | Were transcripts returned to participants for comment and/or correction? | Transcripts were not returned to participants for comment or correction. Transcripts were audio recorded to ensure accuracy |
| **Domain 3: Analysis and findings** | | |
| Data analysis |  |  |
| 1. Number of data coders | How many data coders coded the data? | Two coders were involved in the data analysis (Jemcov, Rosenberg) |
| 1. Description of the coding tree | Did authors provide a description of the coding tree? | Codes were identified to form a code book, that was then applied to the data in order to identify themes |
| 1. Derivation of themes | Were themes identified in advance or derived from the data? | Themes were derived from the data |
| 1. Software | What software, if applicable, was used to manage the data? | Excel spreadsheet and Microsoft Word were utilized to manage the data |
| 1. Participant checking | Did participants provide feedback on the findings? | No |
| Reporting |  |  |
| 1. Quotations presented | Were participant quotations presented to illustrate the themes/findings? Was each quotation identified? | Yes, quotations were used. Quotations were not identified. |
| 1. Data findings consistent | Was there consistency between the data presented and the findings? | Yes |
| 1. Clarity of major themes | Were major themes clearly presented in the findings? | Yes |
| 1. Clarity of minor themes | Is there a description of diverse cases or discussion of minor themes? | Not applicable to the current study |
