# Supplementary material for: Short Report: Barriers and facilitators to parents' implementation of a transdiagnostic eHealth sleep intervention for children with neurodevelopmental disorders
Source: Front Sleep. 2023 Jun 1;2:1143281. doi: 10.3389/frsle.2023.1143281 (PMC12713910; doi:10.3389/frsle.2023.1143281)
Supplement: Supplementary file 2 [file Data_Sheet_1.pdf]

Interview Guide

Interviews were conducted by phone or by online videoconferencing software (Blackboard Collaborate). The interviews were be transcribed and coded qualitatively for key themes. Note that where "[ADHD/ASD/CP/FASD]" is written, parents were asked about the disorder that their child has.

Interviewer (I): Thank you for completing the intervention and participating in this usability study! As you know, your feedback in this study is directly contributing to how we develop and modify the original *Better Nights, Better Days* into an intervention for children with neurodevelopmental disorders. Before we begin, do you have any questions?

Implementation
We're very interested in learning about any factors that could affect parents' ability to use the intervention. I'll ask you some questions about factors that may have affected your experience with and ability to carry out the intervention.

* Note – [*Italicized/bracketed]* = construct of interest for researchers (not to be shared with parents)

1. What, if anything, *positively* affected your motivation and ability to participate in and complete the intervention? (Prompt: What kept you going?)
2. What, if anything, *negatively* affected your motivation and ability to participate in and complete the intervention? (Prompt: What kept you going in spite of this?)
3. Did you end up wanting or needing extra support with the intervention? What did you do? *[Support]*
4. Before you did the intervention, what did you believe about how sleep disorders in children with [ASD/ADHD/FASD/CP] are caused and whether they can be treated? Have your beliefs changed since completing the intervention? *[Sleep-related beliefs/attitudes – causality, treatability]*
5. Before you did the intervention, what did you believe about your own ability to treat your child's sleep problems? Has anything changed since completing the intervention? *[Sleep-related beliefs/attitudes – locus of control]*
6. How has your experience of caring for your child's sleep problems affected your perception of yourself as a parent? How do your child's sleep problems make you feel as a parent? Did the intervention change how you feel about this? *[Parenting self-efficacy/competence]*
7. How did doing and carrying out the intervention affect the rest of your family (e.g., spouse, other children, others living in home)? (Prompt: Positively? Negatively?) *[Broader psychosocial impact]*
8. Did your child's [ADHD/ASD/CP/FASD] symptoms or needs have an effect on your ability to participate in the intervention and implement strategies, and if so, how? *[NDD-specific*]
9. What else affected your ability to participate in and complete the intervention with your child? *[Open-ended*]

Thank you for taking the time to participate in this interview! We appreciate your feedback, which will be invaluable in helping us develop *Better Nights, Better Days for*

Children with Neurodevelopmental Disorders. Please contact me by email at sleepndd@dal.ca if you have any questions.
